# Downregulation of Circular RNA PSEN1 ameliorates ferroptosis of the high glucose treated retinal pigment epithelial cells via miR-200b-3p/cofilin-2 axis

**DOI:** 10.1080/21655979.2021.2010369

**Published:** 2021-12-14

**Authors:** Zhaoliang Zhu, Peng Duan, Huping Song, Rongle Zhou, Tao Chen

**Affiliations:** Ophthalmology Department, Xi’an People’s Hospital, Shaanxi Eye Hospital, Xi’an City, China

**Keywords:** Diabetic retinopathy, ferroptosis, circ-PSEN1, miR-200b-3p, CFL2

## Abstract

Ferroptosis is a form of programmed cell death that participates in the progression of numerous diseases. Long noncoding RNAs (lncRNAs) are dysregulated in diabetic retinopathy (DR). However, the role of lncRNAs in DR-induced ferroptosis is unclear. Adult retinal pigment epithelial cell line-19 (ARPE19) cells were treated with a high concentration of glucose (high glucose, HG) to mimic DR *in vitro*. The intracellular contents of glutathione, malondialdehyde, and ferrous ions were analyzed using the corresponding kits. The MTT assay was performed to measure the cell survival rate, and cell death was determined using propidium iodide and terminal deoxynucleotidyl transferase dUTP nick end labeling staining assays. Western blotting was conducted to detect the protein levels of GPX4, SLC7A11, and TFR1. The targeting relationships were verified using luciferase reporter and RNA pull-down assays. circ-PSEN1 was upregulated in HG-treated ARPE19 cells and showed high resistance to RNase R and Act D. Inhibition of circ-PSEN1 in ARPE19 cells ameliorated the ferroptosis induced by HG was ameliorated, as evidenced by changes in the ferroptosis-related biomarkers/genes and decreased cell death. Subsequently, circ-PSEN1 acted as a sponge for miR-200b-3p. Inhibition of miR-200b-3p partially reversed the effects of circ-PSEN1 on ferroptosis. Furthermore, cofilin-2 (*CFL2*) was the target gene of miR-200b-3p, and it abrogated the inhibitory effect of miR-200b-3p on ferroptosis. Taken together, the findings indicate that knockdown of circ-PSEN1 can mitigate ferroptosis of ARPE19 cells induced by HG via the miR-200b-3p/CFL2 axis.

## Introduction

Diabetic retinopathy (DR) is a common microvascular complication of diabetes, which can cause specific pathologic changes in the fundus. DR is one of the four major blinding diseases [1,2]. Approximately 85% of patients with DR have a history of type 2 diabetes. By 2025, the number of diabetic patients with different degrees of fundus disease is estimated to reach 120 million [3]. Moreover, the extension of the course of diabetic patients will increase the incidence of DR. The main clinical manifestations of DR are retinal vascular diseases, hemorrhagic spots and cotton wool spots in the fundus, macular edema, retinal detachment, and optic neuropathy [4]. According to whether retinal neovascularization exists, DR is classified into non-proliferative diabetic retinopathy and proliferative diabetic retinopathy [5]. Currently, the mechanism of DR is inadequately understood. The main pathophysiological mechanisms include high glucose (HG), oxidative stress, and inflammation, which are activated by high glucose-induced polyols, protein kinase C, advanced glycation end products, renin-angiotensin, and hypoxia [6–8]. Controlling the progression of DR has become one of the most pivotal goals to improve the quality of life of diabetic patients.

Circular RNAs (circRNAs) are a type of noncoding RNA derived from exons of protein-coding genes through the back-splice mechanism [9–16]. The structure of circRNA is a closed loop without 5′-3′ polarity or polyadenylated tails. circRNAs are highly conserved and abundant in human cells [17]. Previous research demonstrated noncoding RNAs participated in various biochemical pathways related to the retinal cell death, including oxidative stress, lipid metabolism, melanin synthesis, and so on [18]. In addition, several studies have reported that circRNAs act as competing endogenous RNAs to sponge the target microRNA (miRNA) and consequently regulate mRNA expression in DR [19]. For instance, downregulation of circDNMT3B contributes to vascular dysfunction in DR by regulating the miR-20b-5p/BAMBI axis [20]. The hsa_circ_0041795 circRNA is enhanced in DR. Conversely, knockdown of hsa_circ_0041795 reportedly promoted miR-646 expression and further inhibited vascular endothelial growth factor (VEGF) C [21]. A previous expression profiling of circRNAs in the vitreous humor of DR and non-diabetes mellitus patients revealed that circ_0008521 (circ-PSNE1) was significantly upregulated in DR patients.

The regulatory effect of circ-PSEN1 on ferroptosis is unknown. In the present study, we aimed to investigate the functions of circ-PSEN1 in ferroptosis in DR with the aim of identifying a potential novel therapeutic target for DR. We hypothesized that circ-PSEN1 relieved DR development via the miR-200b-3p/CFL2 axis.

## Materials and methods

### Cell culture and transfection

The adult retinal pigment epithelial cell line-19 (ARPE19) was purchased from ATCC (cat no. SNL-227). The cells were thawed and then divided into control (normal glucose, NG) and HG groups according to a previous study [22]. The glucose concentrations of Dulbecco’s modified Eagle’s medium (DMEM) were adjusted to 5.5 mM (control) and 25 mM (HG). The cells were cultured in DMEM supplemented with 10% fetal bovine serum (FBS), 100 U/ml streptomycin, and 100 U/ml penicillin (all from ChemeGen) at 37°C in the presence of 5% CO_2_ for 48 hours. After the cell confluence reached 70%–80%, the cells were transfected with small interfering RNA of circ-PSEN1 (si-circ-PSEN1), miR-200b-3p inhibitor, cofilin-2 (CFL2)/pcDNA3.1, and their negative controls (all designed and synthesized by Hanheng Bioengineering Co., Ltd.) using Lipofectamine 3000 (Thermo Fisher Scientific) according to the manufacturer’s protocol.

### Ribonuclease R (RNase R) treatment and RNA degradation assay

The RNA stability was measured according to a previous study [23]. The RNA (1 μg) was incubated with 3 U/μg RNase R or mock (Epicenter) at 37°C for 20 min to remove the linear RNAs. After the treatment, RT-qPCR was used to analyze the level of circ-PSEN1 and PSEN1.

The cells in logarithmic phase were collected and incubated with 0.5 μM actinomycin D (Act D) for 0, 4, 8, 12, and 24 h. After incubation, the cells were collected and lysed to measure the RNA expression levels of circ-PSEN1 and PSEN1 by RT-qPCR.

### Real-time quantitative polymerase chain reaction (RT-qPCR) assay

The RT-qPCR assay was performed as described by Mayu et al. [24]. The total RNA of the cells was extracted by TRIzol (Invitrogen). The RT-qPCR was conducted followed the One-Step qRT-PCR UltraMix (SYBR Green) Kit’s manual (LM-0051; LMAIBio Co., Ltd) on the ABI 7900 PCR System. The relative expression levels were calculated according to the 2^−ΔΔCt^ method. β-actin and U6 were used as internal references, the primer sequences were list in Table 1.

**Table 1.** Primer sequence

	Forward	Feverse
circ-PSEN1	5ʹ-GAGGACAACCACCTGAGCA-3’	5ʹ-TAAGGACCGCAAAGGCTG-3’
PSEN1	5ʹ-GGGAGCCATCACATTATTC-3’	5ʹ-CCTGTGACAAACAAATTATCAG-3’
GPX4	5ʹ-TAAGAACGGCTGCGTGGTGAAG-3’	5ʹ-AGAGATAGCACGGCAGGTCCTT-3’
SLC7A11	5ʹ-GCTGTGATATCCCTGGCATT-3’	5ʹ-GGCGTCTTTAAAGTTCTGCG-3’
TFR1	5ʹ-ACCATTGTCATATACCCGGTTCA-3’	5ʹ-CAATAGCCCAAGTAGCCAATCAT-3’
miR-200b-3p	5ʹ-GCGCTAATACTGTCTGGTAA-3’	5ʹ-CAGTGCGTGTCGTGGAGT-3’
CFL2	5ʹ-GGACCGTTCGACACTGGAGA-3’	5ʹ-AATGGACTGAGCTGGAGAAATGG-3’
β-actin	5ʹ-GCCGGGACCTGACTGACTAC-3’	5ʹ-TTCTCCTTAATGTCACGCACGAT-3’
U6	5ʹ-GCTTCGGCAGCACATATACTAAAAT-3’	5ʹ-CGCTTCACGAATTTGCGTGTCAT-3’

### MTT assay

The cell viability was measured as described by Kumar et al. [25]. The cells were resuspended and inoculated into the 96-well plate. According to the manual of MTT Cell Proliferation and Cytotoxicity Assay Kit (C0009S; Beyotime Biotechnology Co., Ltd.), MTT solution (10 μl) was added to each well of the plate and incubated with the cells at 37°C for 4 h. Next, 100 μl dimethyl sulfoxide was injected to each well to dissolve the formazan crystal. The absorbance values were determined with a microplate reader at the wavelength of 490 nm.

### Western blot analysis

As described by Lakshmi et al. [26]. The proteins were extracted by pre-chilled RIPA lysis buffer (P0013 C; Beyotime) for 30 min then BCA Protein Concentration Assay Kit (E-BC-K168-M; Elabscience Biotech Co., Ltd.) was used to quantify the proteins. The proteins were separated by 10% SDS-PAGE at 120 V for 1.5 h. Subsequently, the separated proteins were transferred onto the PVDF membranes (Millipore) at 200 mA for 2 h. The membranes were then blocked with BSA buffer (WB6015; Biotechwell Co., Ltd.) for 1 h. Anti-GPX4 (1:300; abx102448; Abbexa), anti-SLC7A11 (1:500; 70 R-6800; Fitzgerald), anti-TFR1 (1:200; BS1620; Bioworld) and anti-GAPDH (1:2000; 70 R-32845; Fitzgerald) primary antibodies were incubated with the PVDF membrane at 4°C overnight. GAPDH was used as an internal reference. The next day, the membranes were incubated with a mouse anti human IgG secondary antibody (1:3000; 10 R-2045; Fitzgerald) at room temperature for 2 h. Finally, protein bands were visualized using an ECL system (Thermo Fisher Scientific, Inc.).

### Detection of the ferroptosis biomarkers

The cells were lysed and centrifuged for detecting the amounts of GSH, MDA and Fe^2+^. The amounts of GSH (E-BC-K030-S; Elabscience), MDA (JL11466-48 T; Shanghai Jianglai industrial Co., Ltd.), and Fe^2+^ (E-BC-K304-S; Elabscience) were analyzed by the corresponding kits according to the manufacturer’s protocols.

### PI staining assay

Cell death was determined using the PI Staining Kit (E607306-0100; Sangon Biotech) according to the manufacturer’s manual. Briefly, the cells were resuspended in PBS at the density of 1 × 10^6^/ml. 95 μl of the cell suspension and 5 μl of PI were incubated on dark room for 5 min. DAPI was used for counterstain. At the end of incubation, the PI-positive cells were photographed under a fluorescent microscope (CKX53; Olympus).

### Terminal deoxynucleotidyl transferase dUTP nick end labeling (TUNEL) staining assay

The cells were fixed on a slide with 4% paraformaldehyde and permeabilized with 0.2% Triton X-100 dissolved in PBS. The enhanced fluorescein isothiocyanate TUNEL Assay Kit (E-CK-A334; Elabscience) was used to stain the cells 4′,6-Diamidino-2-phenylindole (DAPI) was used as a counterstain. The number of TUNEL-positive cells was counted by fluorescence microscopy.

### Dual-luciferase reporter assay

Accroding to a previous study [27], the dual-luciferase reporter vectors of wild type (WT) and mutant (MUT) circ-PSEN1 and CFL2 were constructed by VectorBuilder Co., Ltd. They were co-transfected into ARPE19 cells with the miR-200b-3p mimic/control mimic and incubated for 24 h. Luciferase activity was normalized to *Renilla* luciferase activity. The cells were lysed to detect luciferase activity using a chemiluminescence apparatus.

### RNA pull-down assay

Accroding to a previous study [28], The Pierce™ Magnetic RNA-Protein Pull-Down Kit (20,164; Thermo Fisher Scientific) was used to perform the RNA pull-down assay. The biotinylated miR-200b-3p probe and its control probe were synthesized by BersinBio Co., Ltd. Briefly, the probe (50 pmol) was incubated with 50 μL streptavidin coated magnetic beads at 4°C for 2 h. At the end of incubation, the cells were lysed to release total RNA, and the beads were eluted. After separation, RT-qPCR was used to quantify the relative expression of circ-PSEN1 and CFL2, as described above.

### Statistical analyses

GraphPad Prism (version 8.4.3.686; GraphPad Software, Inc.) was used to analyze the data. Three independent experiments were conducted for each assay. Data are presented as the mean ± SD. Student’s t-test was employed to calculate the statistical differences between the two groups. One way ANOVA followed by Turkey’s post-hoc test was applied for more than three groups. P < 0.05 indicates a statistically significant difference.

## Results

This study demonstrated that circ-PSEN1 and CFL2 were up-regulated and miR-200b-3p was down-regulated in DR. downregulation of circ-PSEN1 ameliorates HG-induced ferroptosis in ARPE19 cells via miR-200b-3p/CFL2 and may be a novel therapeutic target for DR.

### Circ-PSEN1 is stable and elevated in the HG-treated ARPE19 cells

**Figure 1.**
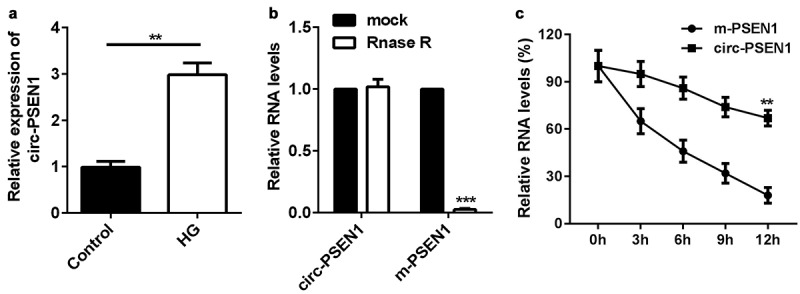
Elevation of circ-PSEN1 by HG treatment in ARPE19 cells. (a) RT-qPCR determined expression levels of circ-PSEN1 in the ARPE19 cells treated with normal level glucose and HG for 48 hours. (b) The RNA levels of circ-PSEN1 and linear PSEN1 were measured by RT-qPCR after RNase R was incubated with the total cellular RNA. Mock treated cells were used as the negative control. (c) Relative RNA levels of circ-PSEN1 and PSEN1 were recorded 0, 4, 8, 12, 24 h after the cells were treated by actinomycin D. Data are representative of three experiments. **P < 0.01; ***P < 0.001. HG, high glucose; ARPE19, adult retinal pigment epithelial cell line-19; Act D, actinomycin D

The expression levels of circ-PSEN1 were detected in HG or NG treated ARPE19 cells using PT-qPCR. As shown in Figure 1(a), HG treatment significantly elevated the level of circ-PSEN1. Next, we investigated the stability of circ-PSEN1. RNase R notably decreased the RNA level of linear PSEN1, whereas circ-PSEN1 was not affected by RNase R (Figure 1(b)). Moreover, circ-PSEN1 exhibited stronger resistance to Act D relative to mRNA PSEN1. Its half-life was longer than that of the linear PSEN1 (Figure 1(c).

### Si-circ-PSEN1 regulates intracellular concentrations of glutathione (GSH), malondialdehyde (MDA), and ferrous iron of HG-treated cells

**Figure 2.**
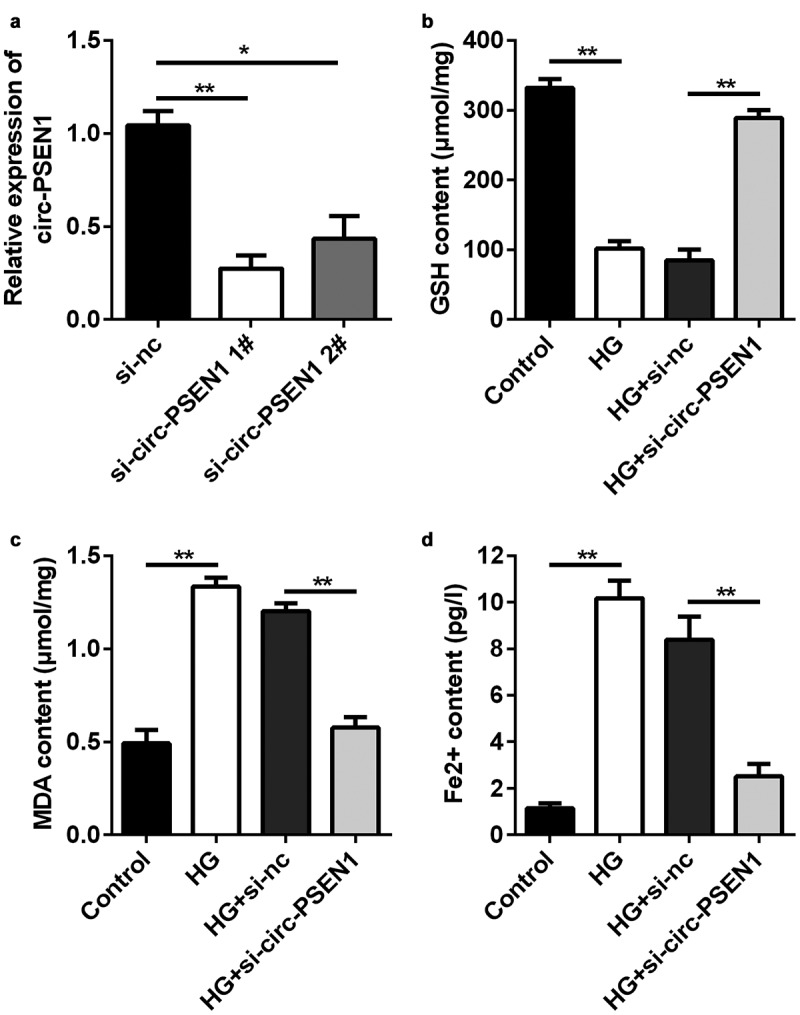
Si-circ-PSEN1 regulates contents of GSH, Fe^2+^, and MDA in the HG-treated ARPE19 cells. (a) The expression levels of circ-PSEN1 when the ARPE19 cells were transfected with si-circ-PSEN1 1# and si-circ-PSEN1 2#. (b) GSH, (c) MDA, and (d) Fe^2+^ contents were examined in the HG-treated cells 48 h after transfection with si-circ-PSEN1 using the corresponding kits. Data are representative of three experiments; *P < 0.05; **P < 0.01. HG, high glucose; ARPE19, adult retinal pigment epithelial cell line-19; GSH, glutathione; MDA, malondialdehyde

Since circ-PSEN1 was upregulated in HG-treated ARPE19 cells, we transfected si-circ-PSEN1 into the cells to observe changes in cellular phenotypes. qPCR results indicated that both siRNAs targeting circ-PSEN1 reduced the expression level of circ-PSEN1 (Figure 2(a)). The GSH concentration was markedly reduced after HG treatment. In contrast, si-circ-PSEN1 enhanced the concentration of GSH in HG-treated cells (Figure 2(b)). HG also increased MDA concentration. Si-circ-PSEN1 partially abrogated the effect of HG on MDA (Figure 2(c)). Similarly, HG triggered the enhancement of intracellular ferrous iron, but si-circ-PSEN1 ameliorated this enhancement (Figure 2(d)).

### Knockdown of circ-PSEN1 suppresses ferroptosis of HG-treated ARPE19 cells

**Figure 3.**
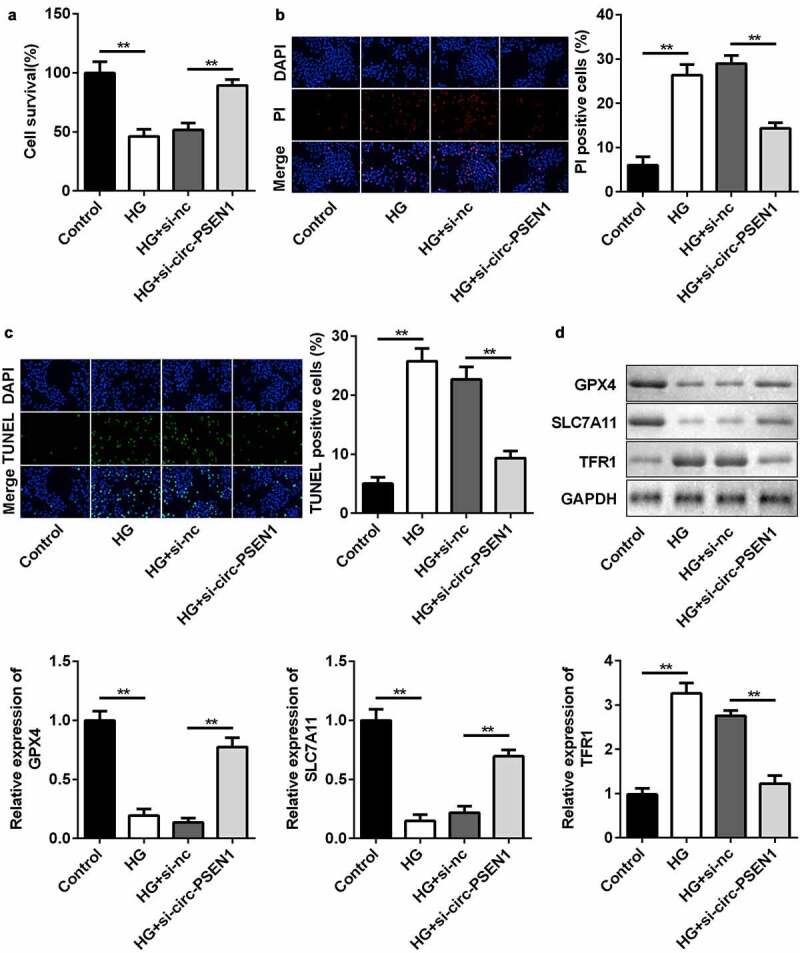
Downregulation of circ-PSEN1 alleviates ferroptosis of HG-induced ARPE19 cells. (a) The MTT assay was used to assess the cell survival rate of the cells treated with HG and si-circ-PSEN1 for 24 h. (b) PI positive cells were photographed and counted by fluorescence microscopy. DAPI was used for counter staining. The ARPE19 cells were treated with HG and si-circ-PSEN1 for 24 h. (c) TUNEL positive cells were captured and counted by fluorescence microscopy. The ARPE19 cells were treated with HG and si-circ-PSEN1 for 24 h. (d) The protein expressions of GPX4, SLC7A11, and TFR1 were detected by Western blotting 24 h after the indicated treatments. Data are representative of three experiments; **P < 0.01. HG, high glucose; ARPE19, adult retinal pigment epithelial cell line-19; PI, propidium iodide; DAPI, 4′,6-diamidino-2-phenylindole; TUNEL, TdT mediated dUTP Nick End Labeling

The levels of ferroptosis biomarkers were regulated by si-circ-PSEN1. Next, we detected cell death and mRNA expression of ferroptosis-related genes in ARPE19 cells after HG and si-circ-PSEN1 treatments. HG significantly reduced cell survival rate. The survival rate of the cells treated with both HG and si-circ-PSEN1 increased (Figure 3(a)). Cell death was analyzed by propidium iodide (PI) and TUNEL staining assays. As shown in Figure 3(b) and (c), si-circ-PSEN1 reduced PI-positive cells or TUNEL-positive cells in HG-treated cells. Furthermore, the protein/mRNA expression of GPX4 and SLC7A11 was inhibited, whereas the expression of TFR1 was promoted by HG. This effect of HG was reversed by si-circ-PSEN1 (Figure 3(d)).

### miR-200b-3p is directly targeted by circ-PSEN1

**Figure 4.**
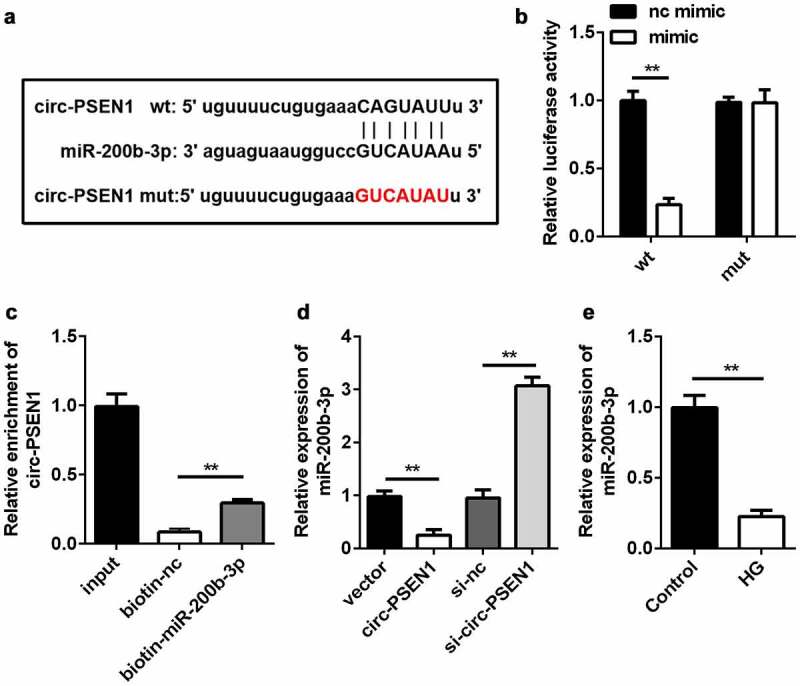
miR-200b-3p is the target of circ-PSEN1. (a) The sequences of circ-PSEN1 WT, circ-PSEN1 MUT, and miR-200b-3p. (b) Luciferase activities of the ARPE19 cells were analyzed 24 h after the indicated treatments. (c) The levels of circ-PSEN1 enriched by negative control and biotin-labeled miR-200b-3p. (d) The expression levels of miR-200b-3p of the cells transfected with circ-PSEN1, si-circ-PSEN1, or their control. (e) The expression level of miR-200b-3p in the HG-treated ARPE19 cells. Data are representative of three experiments; **P < 0.01. HG, high glucose; ARPE19, adult retinal pigment epithelial cell line-19; WT, wild type; MUT, mutant type

Subsequently, we searched for the target miRNAs of circ-PSEN1. Given that miR-200b-3p was predicted to be the target of circ-PSEN1 and was previously shown to be downregulated in DR [29], we chose miR-200b-3p for further studies. The sequences of miR-200b-3p, circ-PSEN1 WT, and circ-PSEN1 MUT are shown in Figure 4(a). The sequences of circ-PSEN1 WT and circ-PSEN1 MUT were cloned into luciferase vectors. The miR-200b-3p mimic decreased luciferase activity in cells transfected with the circ-PSEN1 WT vector. However, no change was observed in the circ-PSEN1 MUT group (Figure 4(b)). The RNA pull-down results showed that miR-200b-3p enriched a markedly higher level of circ-PSEN1 (Figure 4(c)). These data verified the targeting relationship between miR-200b-3p and circ-PSEN1. Moreover, an excessive amount of circ-PSEN1 suppressed miR-200b-3p expression, whereas knockdown of circ-PSEN1 had the opposite effect (Figure 4(d)). In HG-treated ARPE19 cells, miR-200b-3p was downregulated (Figure 4(e)).

### Inhibiting miR-200b-3p abrogates effects of si-circ-PSEN1 on cell survival and ferroptosis

**Figure 5.**
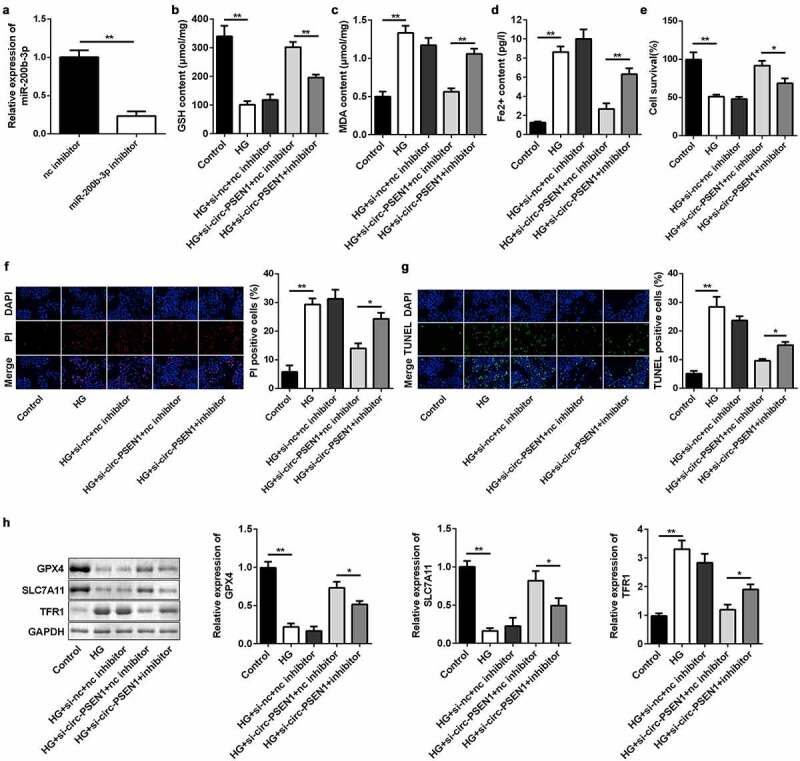
Inhibition of miR-200b-3p abrogates the effects of si-circ-PSEN1 on ferroptosis. (a) The expression level of miR-200b-3p in ARPE19 cells transfected with miR-200b-3p inhibitor. (b) GSH contents, (c) MDA contents, and (d) Fe^2+^ in HG-treated cells 48 h after transfection. (e) The MTT assay was used to assess the cell survival rate of the cells treated with HG and the indicated plasmids after 24 h. (f) PI positive cells and (g) TUNEL positive cells were photographed and counted by fluorescence microscopy. (h) Protein expressions of GPX4, SLC7A11, and TFR1 detected by Western blotting assay 24 h after the indicated treatments. Data are representative of three experiments; *P < 0.05; **P < 0.01. HG, high glucose; ARPE19, adult retinal pigment epithelial cell line-19; GSH, glutathione; MDA, malondialdehyde; PI, propidium iodide; DAPI, 4ʹ,6-diamidino-2-phenylindole; TUNEL, TdT mediated dUTP Nick End Labeling

We conducted rescue experiments in ARPE19 cells to further examine the targeting relationship between circ-PSEN1 and miR-200b-3p at the epigenetic level. The miR-200b-3p inhibitor downregulated the level of endogenous miR-200b-3p (Figure 5(a)). As expected, transfection of miR-200b-3p inhibitor markedly decreased the concentration of GSH and increased the MDA and ferrous ion contents (Figure 5(b–d)). The miR-200b-3p inhibitor suppressed the elevated cell survival level caused by si-circ-PSEN1 (Figure 5(e)). Conversely, PI- and TUNEL-positive cell numbers were promoted by the miR-200b-3p inhibitor (figure 5(f) and (g)). In addition, the mRNA and protein levels of the anti-ferroptosis genes (GPX4 and SLC7A11) were reduced by miR-200b-3p, whereas TFR1 was promoted (Figure 5(h)).

### *miR-200b-3p targets* CFL2

**Figure 6.**
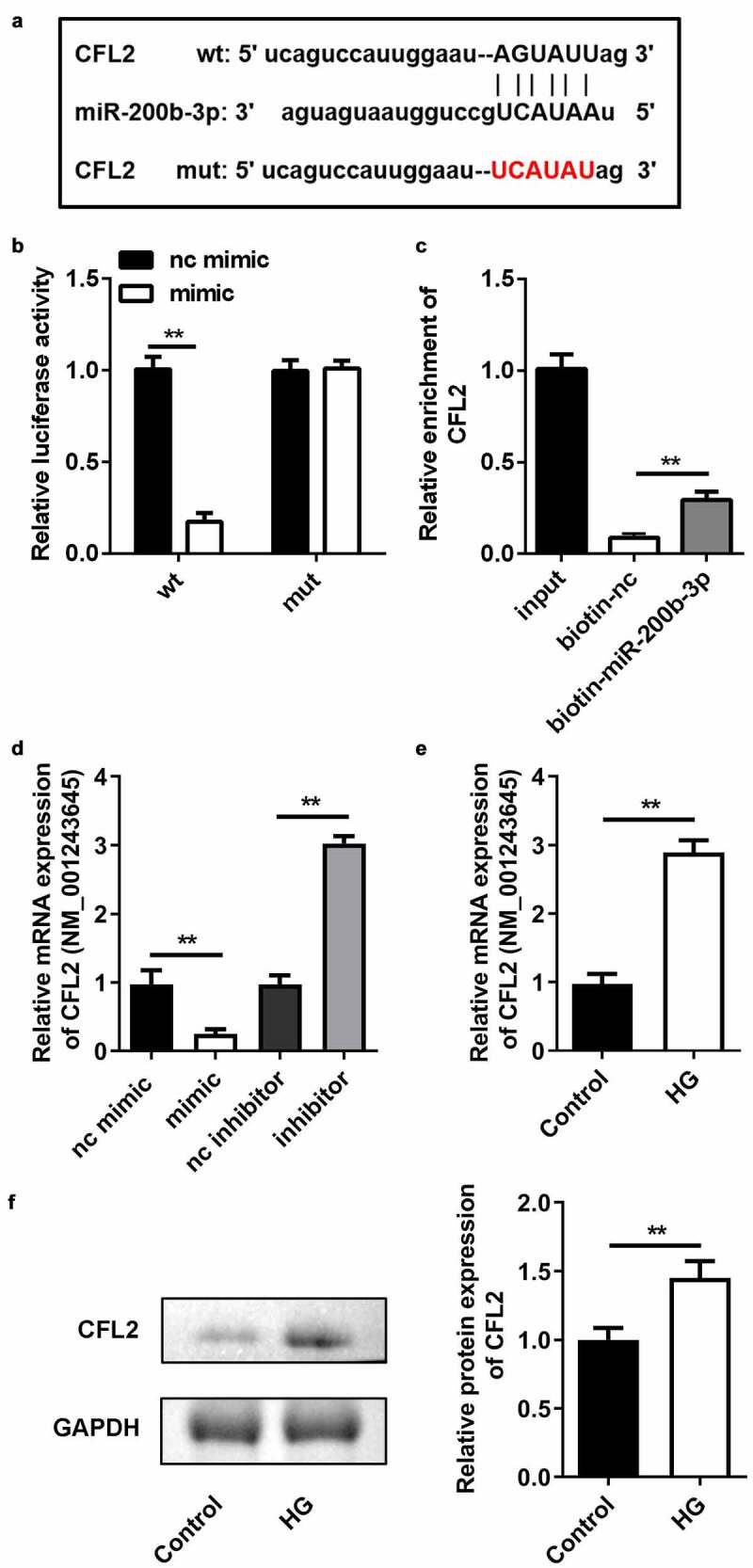
*CFL2* is the target gene of miR-200b-3p. (a) The sequences of CFL2 WT, CFL2 MUT and miR-200b-3p. (b) Luciferase activities of the ARPE19 cells were analyzed 24 h after the indicated treatments. (c) The levels of CFL2 enriched by negative control and biotin-labeled miR-200b-3p. (d) The expression levels of CFL2 of the cells transfected with miR-200b-3p mimic, miR-200b-3p inhibitor, or their control. (e) The expression level of CFL2 in the HG-treated ARPE19 cells. Data are representative of three experiments; **P < 0.01. CFL2, cofilin-2; HG, high glucose; ARPE19, adult retinal pigment epithelial cell line-19; WT, wild type; MUT, mutant type

We used the TargetScan online bioinformatics tool to predict the target gene of miR-200b-3p. CFL was identified as the potential target. Likewise, the luciferase reporter vectors of CFL WT and MUT were co-transfected into ARPE19 cells with the miR-200b-3p mimic (Figure 6(a)). In cells transfected with the CFL2 WT vector, luciferase activity was significantly lower in the miR-200b-3p mimic group. However, there was no change in luciferase activity between the CFL2 MUT group (Figure 6(b)). RNA pull-down results indicated that the biotin-labeled miR-200b-3p enriched higher levels of CFL2 (Figure 6(c)), further verifying the predictive analysis. Next, we found that CFL2 expression level was elevated by miR-200b-3p mimic, but was decreased by miR-200b-3p inhibitor (Figure 6(d)). Furthermore, CFL2 expression was upregulated in HG-treated cells both in mRNA and protein levels (Figure 6(e-f)).

### Rescue of CFL2 reverses the influences of miR-200b-3p on cell survival and ferroptosis

**Figure 7.**
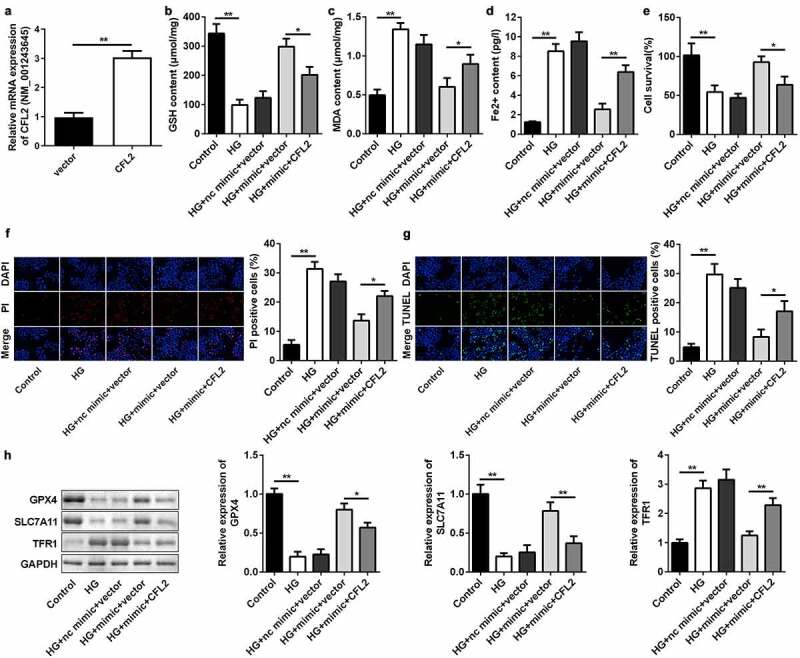
CFL2 reverses the effects of miR-200b-3p on cell ferroptosis. (a) The expression level of CFL2 in the ARPE19 cells transfected with CFL2/pcDNA3.1. (b) GSH, (c) MDA, and (d) Fe^2+^ contents were examined in HG-treated cells 48 h after transfection. (e) The MTT assay was used to assess the cell survival rate of the cells treated with HG and the indicated vectors for 24 h. (f) PI positive cells and (g) TUNEL positive cells were photographed and counted by fluorescence microscopy. (h) Protein expressions of GPX4, SLC7A11, and TFR1 were detected by Western blotting 24 h after the cells underwent the indicated treatments. The mRNA expression levels of their genes were detected by RT-qPCR. Data are representative of three experiments; *P < 0.05; **P < 0.01. CFL2, cofilin-2; HG, high glucose; ARPE19, adult retinal pigment epithelial cell line-19; GSH, glutathione; MDA, malondialdehyde; PI, propidium iodide; DAPI, 4′,6-diamidino-2-phenylindole; TUNEL, TdT mediated dUTP Nick End Labeling

To further explore the relationship between miR-200b-3p and CFL2 in ARPE19 cells, we rescued the level of CFL2 in HG-treated cells transfected with miR-200b-3p. The CFL2 overexpression plasmid was transfected into ARPE19 cells, which dramatically increased the CFL2 level (Figure 7(a)). CFL2 overexpression resulted in a decrease in GSH and increases in MDA and ferrous ions (Figure 7(b–d)). In addition, CFL2 lowered the cell survival rate (Figure 7(e)). Cell death was promoted when CFL2 was upregulated in the cells, as evidenced by the increased PI and TUNEL-positive cells (figure 7(f) and (g)). Finally, we determined the expression of GPX4, SLC7A11, and TFR1. As displayed in Figure 7(h), enhancement of CFL2 suppressed the mRNA and protein expression of GPX4 and SLC7A11. However, TFR1 expression was promoted.

## Discussion

Previous studies demonstrated that circ-PSEN1 is upregulated in DR [30–32]. On the basis of this finding, we intended to further investigate whether circ-PSEN1 functioned in the DR-induced ferroptosis. We found that circ-PSEN1 was promoted in HG-treated ARPE19 cells. Knockdown of circ-PSEN1 increased the cell survival rate and suppressed ferroptosis in HG-treated ARPE19 cells. Subsequently, circ-PSEN1 was verified to sponge miR-200b-3p. Inhibition of miR-200b-3p abrogated the effects of si-circ-PSEN1 on ferroptosis. Additionally, *CFL2* was identified as the target gene of miR-200b-3p, which aggravated HG-induced ferroptosis in miR-200b-3p-overexpressed cells.

Some circRNAs may be dysregulated in DR and are associated with cellular functions. For example, HG induction led to an increase in the circCOL1A2 expression level in human retinal microvascular endothelial cells (hRMECs), and an excessive amount of circCOL1A2 promoted VEGF expression by sponging miR-29b. Deletion of circCOL1A2 suppresses proliferation, migration, angiogenesis, and vascular permeability of hRMECs induced by HG [33]. Furthermore, the level of hsa_circ_0041795 was reportedly enhanced in HG-treated ARPE19 cells, whereas downregulation of hsa_circ_0041795 accelerated proliferation and inhibited apoptosis of HG-induced ARPE19 cells [21]. However, research on circRNAs in ferroptosis has mainly focused on tumors, such as glioma, breast cancer, rectal cancer, and hepatocellular carcinoma [34–37]. Circ-PSEN1 was previously shown to be overexpressed in DR [32]. Thus, we assessed whether circ-PSEN1 participates in DR-induced ferroptosis. We observed that circ-PSEN1 was upregulated in HG-treated ARPE19 cells, consistent with a previous study. In addition, circ-PSEN1 was very resistant to RNase R and actinomycin D treatment, implying a circular nature. Knockdown of circ-PSEN1 promoted cell survival and suppressed ferroptosis in HG-treated ARPE19 cells. These collective findings indicate that inhibiting circ-PSEN1 contributed to the relief of ferroptosis induced by HG treatment, indicating its potential therapeutic function in DR-induced ferroptosis.

CircRNA functions as an endogenous competing RNA for miRNAs. Yu et al demonstrated circ-UBAP2-silenced attenuated the oxidative stress and dysfunctions of the hRMECs stimulated by HG via regulating the miR-589-5p expressions [38]. In the present study, miR-200b-3p was verified to be sponged by circ-PSEN1 in HG-treated ARPE19 cells. Accumulating evidence suggests that miRNAs influence the development of DR. For instance, miR-139-5p promotes cell migration, tube formation, and expression of VEGF protein in HG-treated human retinal microvascular endothelial cells. Silencing miR-139-5p produces the opposite effect [39]. In an *in vivo* study, miR-19a expression levels in retinal ganglion cells of DR rats were enhanced. Inhibition of miR-19a reportedly decreased apoptosis of these cells [40]. In the present study, miR-200b-3p was downregulated in HG-treated ARPE19 cells, similar to a previous report [29]. Furthermore, miR-200b-3p is the target of circ-PSEN1. We transfected the miR-200b-3p inhibitor into ARPE19 cells treated with HG and si-circ-PSEN1. As expected, miR-200b-3p inhibitor reversed the effects of si-circ-PSEN1, suggesting that miR-200b-3p mechanically interacts with circ-PSEN1.

Additionally, we found that *CFL2* was the target gene of miR-200b-3p. Yu et al. [41] found that knockdown of CFL2 in nasopharyngeal carcinoma cells notably reduced proliferation, but facilitated apoptosis and radiosensitivity of the cells. Moreover, *CFL2* acts as a target gene of miR-369-3p in prostate cancer. Overexpression of CFL2 exacerbated the carcinogenic behavior of prostate cancer cells [42]. However, CFL2 has rarely been investigated in DR-induced ferroptosis. In this study, we overexpressed CFL2 in ARPE19 cells. This overexpression resulted in the aggravation of ferroptosis, which abrogated the effects of miR-200b-3p.

In conclusion, downregulation of circ-PSEN1 ameliorates HG-induced ferroptosis in ARPE19 cells via miR-200b-3p/CFL2 and may be a novel therapeutic target for DR.

## Data Availability

All the data is available from the corresponding author due to the reasonable request.

## Highlights

- Circ-PSEN1 is elevated in DR
- miR-200b-3p is directly targeted by circ-PSEN1
- miR-200b-3p targets CFL2
- circ-PSEN1 relieves DR development via the miR-200b-3p/CFL2 axis.
